# Sclerema neonatorum in a premature newborn: A case report

**DOI:** 10.1002/ski2.255

**Published:** 2023-05-25

**Authors:** Rima Alhalabi, Riham Salloum, Dima Alhalabi, Youlla Oun, Julia Abudeeb, Judy Kikhia, Manal Mouhamad

**Affiliations:** ^1^ Department of Dermatology and Venereology Damascus University Damascus Syria; ^2^ Department of Pediatrics Children's University Hospital Faculty of Medicine Damascus University Damascus Syria; ^3^ Faculty of Medicine Syrian Private University Damascus Syria

## Abstract

Sclerema neonatorum (SN) is a rare condition of neonatal panniculitis with a poor prognosis and a high fatality rate. It clinically presents as hardening of the skin and subcutaneous adipose tissue extending throughout the body, sparing the fat‐free soles, palms, and genitalia. SN typically affects gravely ill, preterm neonates in the first week of life and diagnosis is often clinical. We report a case of an eight‐day‐old premature infant diagnosed with early‐onset neonatal sepsis who presented with clinical and histopathological features of SN. Despite early treatment of the sepsis with intravenous antibiotics and the SN with a topical corticosteroid cream and moisturiser, the infant died on the twelfth day of life.

## Graphical abstract

Sclerema Neonatorum is a rare skin condition with a poor prognosis affecting neonates. We report a case of a preterm neonate with clinical and histologic features of Sclerema Neonatorum, with a detailed description of the patient&apos;s initial presentation, disease progression, diagnosis, and treatment.

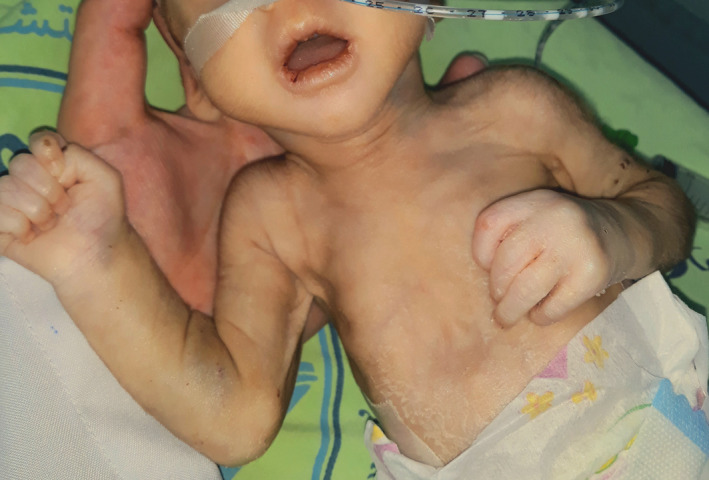

## 1 INTRODUCTION

Sclerema neonatorum (SN) is a rare form of neonatal panniculitis that manifests as generalised cutaneous and subcutaneous adipose tissue induration. The sclerosis can progress to affect both feeding and respiratory functions and become life‐threatening.^1^ The exact pathophysiology of this condition remains unknown; however, several theories are proposed, including abnormal fat metabolism, hardening of the fat with falling body temperatures triggered by shock, or a consequence of systemic disease.^1^ We present the case of a premature newborn male hospitalised for early‐onset neonatal sepsis who developed SN. To the best of our knowledge, this is the first reported case of SN in the Syrian dermatologic literature.

## 2 CASE PRESENTATION

A premature eight‐day‐old male infant presented to the outpatient clinic at Damascus Dermatology Hospital for evaluation of progressive skin sclerosis. The patient was delivered at 32 weeks of gestation by vaginal delivery at home to a healthy gravida 3 para 3 mother. The pregnancy and delivery were uncomplicated, and the mother had no known illnesses or infections at the time of delivery.

On his first day of life, the infant was admitted to the neonatal intensive care unit at Damascus University Children's Hospital due to high fever and dyspnea. Physical examination showed a weight of 1500 g, axillary temperature of 39°C, heart rate of 190 bpm, respiratory rate of 45/min, and oxygen saturation of 92% at room air. No congenital anomalies were noted, and the rest of the exam was normal. Blood and urine samples were collected and sent for analysis. Forty‐eight hours after hospital admission, the patient developed skin hardening of the lower limbs that gradually progressed to the rest of the body and face. Blood analysis revealed a total leukocyte count of 23900/mm^3^ with 75% neutrophil dominance, a platelet count of 183000/mm^3^, and a hemoglobin value of 13.3 g/dl. Serum sodium was 140 mmol/L, serum potassium was 5.5 mmol/L, urea was 25 mg/dl, creatinine was 0.9 mg/dl, and procalcitonin was 19 μg/L. Blood and urine cultures did not grow any organisms, and chest x‐ray and CSF studies showed no abnormalities. A provisional diagnosis of early‐onset neonatal sepsis was made, and the patient was started on intravenous fluids, intravenous vancomycin and ceftazidime, and a nasogastric tube for feeding was placed.

**FIGURE 1:**
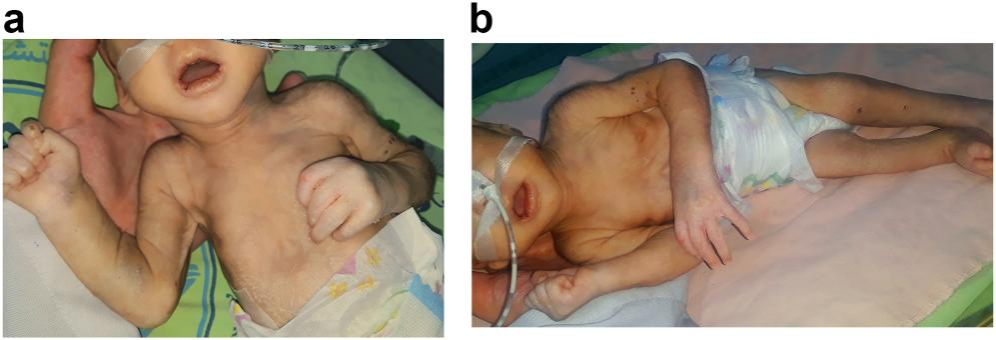
Clinical presentation of the patient at 8 days of age with woody induration of the skin involving the entire body, sparing both nipples, back, palms, soles, and the genital area (a, b).

**FIGURE 2:**
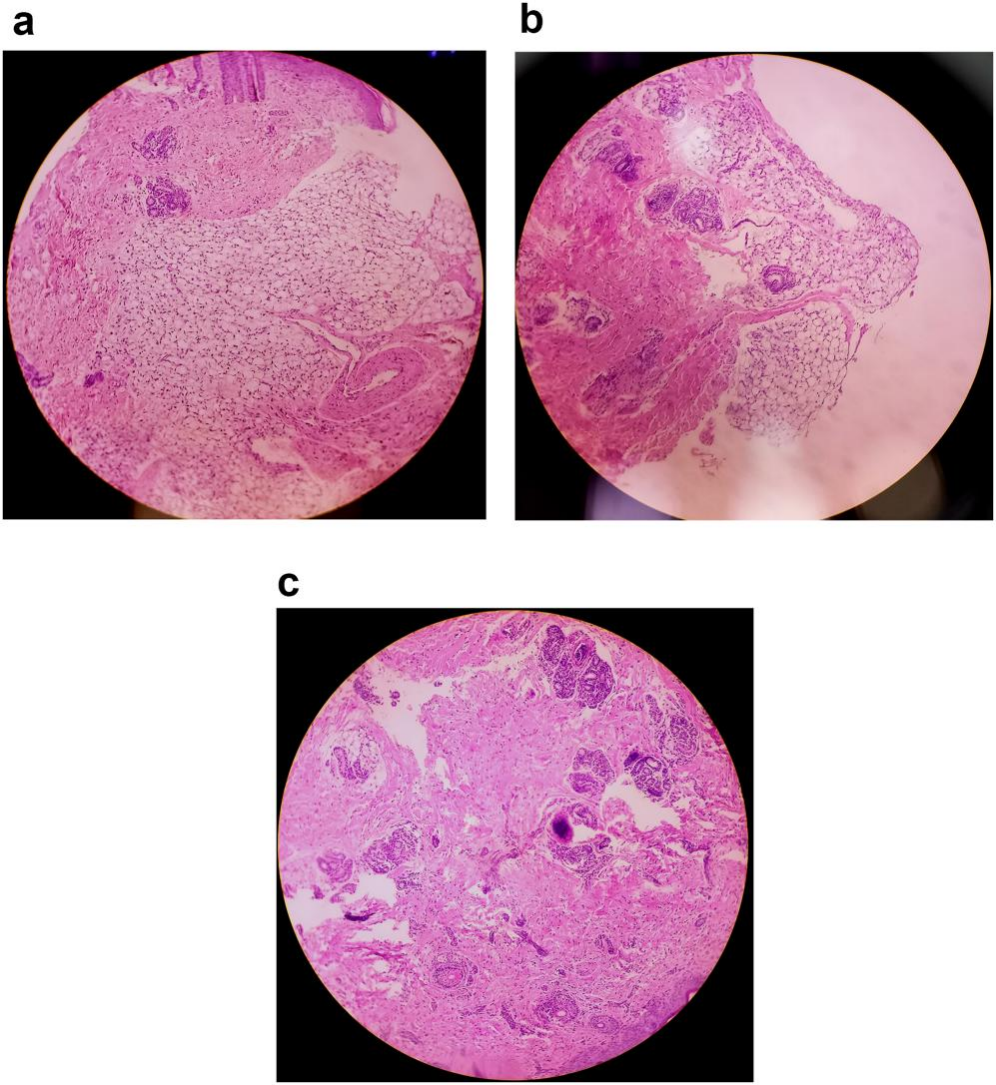
Histologic findings (H&E, x100). (a) Extensive fibrosis surrounding the fat lobules with dense dermal collagen deposition. (b) Sparse lymphocytic inflammatory infiltration. (c) Entrapment of the dermal adnexa between the collagen fibres.

On his eighth day of life, the infant was brought to the Damascus Dermatology Hospital outpatient clinic. Upon examination, woody indurated skin was observed over the entire body, sparing both nipples, back, palms, soles, and the genital area (Figure 1). A skin biopsy of a sclerotic lesion on the thigh revealed extensive fibrosis surrounding the fat lobules with dense dermal collagen deposition (Figure 2a), sparse lymphocytic inflammatory infiltration (Figure 2b), and entrapment of the dermal adnexa between the collagen fibres (Figure 2c). Based on the clinical and histologic findings, a diagnosis of SN was made on day 10 of life. The infant was treated with a topical mixture of 70% Vaseline and 30% mometasone twice daily, and the antibiotics were continued for the treatment of sepsis. Despite all treatments, the infant died at 12 days of life.

## 3 DISCUSSION

Sclerema Neonatorum is an inflammatory skin condition characterised by gradual yet pronounced hardening of the skin. It is principally documented in premature, low‐birth‐weight infants in their first few days of life who frequently have additional risk factors such as sepsis, respiratory and GI tract infections, and birth defects.^1^ Fewer cases have emerged in the last decade due to advanced perinatal intensive care^2^, ^3^; nevertheless, novel cases are still reported, often in precarious perinatal circumstances.^4^ SN carries a dismal prognosis, with mortality rates as high as 98% in most cases.^5^ Our patient characteristically presented on the third day of life and was premature, low‐birthweight, and diagnosed with early‐onset neonatal sepsis. Although he had no known congenital disabilities, the aforementioned clinical findings, as well as his home birth — most likely in unsanitary conditions— are all risk factors for the development of SN.

Clinically, SN presents with progressive sclerosis in which the epidermis firmly adheres to the underlying tissue, making pinching or stretching the skin difficult. Joint contractures can develop, resulting in decreased mobility and ischemia of the extremities.^6^ The taut skin impairs thoracic wall movement during respirations and affects the GI system as well.

Given that SN can present similarly to other less fatal diseases such as subcutaneous fat necrosis of the newborn (SCFNN), scleredema, and stiff skin syndrome clinically, a biopsy is often preferred to confirm the diagnosis, as prognosis and treatment differ. Archetypal pathological findings include necrosis of subcutaneous fat tissue with needle‐shaped cleft formation within fat cells, thick fibrosis surrounding fat lobules, and sparse inflammatory infiltration without granulomatous changes.^3^, ^7^

In our case, the infant presented with clinical manifestations suggestive of SN, however, a biopsy was performed in order to establish the proper diagnosis. Although neither fat necrosis nor the formation of needle‐shaped clefts was noted on pathology, the biopsy nonetheless supported our diagnosis.

It is imperative to differentiate SN from SCFNN, as the former is often fatal. SCFNN manifests as localised red or violaceous areas of the skin in a healthy full‐term neonate, with fat necrosis and extensive inflammatory infiltrate on histology.^8^, ^9^ The disease is self‐limiting, and treatment is supportive. Contrarily, our patient was premature with generalised skin thickening, a sparse inflammatory infiltrate on skin biopsy, and required more serious medical intervention.

Scleredema of the newborn is another possible differential diagnosis given that it affects preterm neonates in the first week of life. However, it differs in that the skin and subcutaneous tissue are edematous and easily pitted and is often self‐limiting with a low mortality rate.^1^

SN should also be distinguished from stiff skin syndrome (SSS) in which infants present with a localised, severely sclerotic skin lesion, often on the thighs or buttocks.^10^ The overlying skin may demonstrate hypertrichosis and hyperpigmentation. Histopathologic features include increased dermal mucin, giant fibroblasts, thickening of the fascia, and absent inflammatory infiltrate.^10^ Notably, SSS does not threaten vital functions, and treatment revolves around preserving mobility.

No definite guidelines are set for the management of SN; some cases have reported the use of systemic corticosteroids which limited the extension of the lesions with no positive effect on overall mortality,^11^, ^12^, ^13^ while others treated a healthy full‐term baby with topical moisturizers without the use of systemic medications.^5^ One case report utilised intravenous immunoglobulin in a neonate with SN that subsequently developed septicemia, and although the IVIG did not prevent mortality, it led to a significant improvement in clinical symptoms, warranting further research into the efficacy of such treatment.^14^ Unfortunately, given the cost and availability of such treatment, we were unable to implement it into our treatment plan. Shrestha, et. al successfully managed SN in a preterm neonate with systemic hydrocortisone, antibiotics, and intensive care management.^15^ These cases suggest that early management with systemic corticosteroids, along with proper treatment of comorbidities that could be the underlying cause of the development of SN, may be beneficial. Despite receiving early treatment with intravenous antibiotics and a topical corticosteroid which improved his general condition, the baby died, which was predictable given the high overall mortality rate in infants with SN.

## 4 CONCLUSION

Whilst our case is quite typical of SN in its clinical presentation and histopathologic findings, Sclerema neonatorum is still a rare condition that necessitates early recognition and intervention. Our case reflects the importance of considering SN in highly suggestible cases and administering prompt treatment, particularly in areas where perinatal care is inadequate. Given its poorly understood pathogenesis and lack of clear guidelines for diagnosis and treatment, SN unfortunately remains highly fatal in the majority of cases.

## AUTHOR CONTRIBUTIONS

**Rima Alhalabi**: Conceptualization (lead); Writing—original draft (lead). **Riham Salloum**: Conceptualization (equal); Methodology (equal); Writing—original draft (equal). **Dima Alhalabi**: Data curation (equal); Methodology (equal); Writing—original draft (equal). **Youlla Oun**: Data curation (supporting); Methodology (supporting); Writing—original draft (supporting). **Julia Abudeeb**: Methodology (supporting); Writing—original draft (supporting). **Judy Kikhia**: Methodology (equal); Writing—original draft (equal); Writing—review & editing (lead). **Manal Mouhamad**: Supervision (lead); Writing—review & editing (equal).

## CONFLICT OF INTEREST STATEMENT

The authors declare that they have no conflict of interest.

## ETHICS STATEMENT

Approval was obtained from the ethics committee of the Faculty of Medicine at Damascus University. The procedures used in this study adhere to the tenets of the Declaration of Helsinki.

## Data Availability

The authors confirm that the data supporting the findings of this study are available within the article and/or its supplementary materials.
